# Supplementary material for: Interpretable embeddings of speech explain and enhance the brain encoding performance of audio models
Source: PLoS Comput Biol. 2026 Oct 30;22(10):e1014906. doi: 10.1371/journal.pcbi.1014906 (PMC13722164; doi:10.1371/journal.pcbi.1014906)
Supplement: Supplementary file 1 [file pcbi.1014906.s003.pdf]

**S1 Table. List of all questions in question-answering features.**

| Question |
|---|
| 1. Does the sentence describe a personal reflection or thought? |
| 2. Does the sentence contain a proper noun? |
| 3. Does the sentence describe a physical action? |
| 4. Does the sentence describe a personal or social interaction that leads to a change or revelation? |
| 5. Does the sentence involve the mention of a specific object or item? |
| 6. Does the sentence involve a description of physical environment or setting? |
| 7. Does the sentence describe a relationship between people? |
| 8. Does the sentence mention a specific location? |
| 9. Is time mentioned in the input? |
| 10. Is the sentence abstract rather than concrete? |
| 11. Does the sentence express the narrator's opinion or judgment about an event or character? |
| 12. Is the input related to a specific industry or profession? |
| 13. Does the sentence include dialogue? |
| 14. Does the sentence describe a visual experience or scene? |
| 15. Does the input involve planning or organizing? |
| 16. Does the sentence involve spatial reasoning? |
| 17. Does the sentence involve an expression of personal values or beliefs? |
| 18. Does the sentence contain a negation? |
| 19. Does the sentence describe a sensory experience? |
| 20. Does the sentence include technical or specialized terminology? |
| 21. Does the input contain a number? |
| 22. Does the sentence contain a cultural reference? |
| 23. Does the text describe a mode of communication? |
| 24. Does the input include a comparison or metaphor? |
| 25. Does the sentence express a sense of belonging or connection to a place or community? |
| 26. Does the sentence describe a specific sensation or feeling? |
| 27. Does the text include a planning or decision-making process? |
| 28. Does the sentence include a personal anecdote or story? |
| 29. Does the sentence involve a discussion about personal or social values? |
| 30. Does the text describe a journey? |
| 31. Does the input contain a measurement? |
| 32. Does the sentence describe a physical sensation? |
| 33. Does the sentence include a direct speech quotation? |
| 34. Is the sentence reflective, involving self-analysis or introspection? |
| 35. Does the input describe a specific texture or sensation? |
| 36. Is the input about a discovery or realization? |
| 37. Does the sentence include an account of a miscommunication or misunderstanding? |
| 38. Does the sentence include a specific sound or auditory description? |
| 39. Does the sentence use a unique or unusual word? |
| 40. Does the sentence describe a change in a physical or emotional state? |
| 41. Does the sentence describe a moment of relief or resolution of tension? |
| 42. Does the sentence include a conditional clause? |
| 43. Does the sentence reference a specific time or date? |
| 44. Is the sentence conveying the narrator's physical movement or action in detail? |
| 45. Is there mention of a city, country, or geographic feature? |
| 46. Does the sentence involve an unexpected incident or accident? |
| 47. Does the sentence involve a recount of a social or community event? |
| 48. Does the sentence express a philosophical or existential query or observation? |
| 49. Does the story involve a personal project or creation? |
| 50. Is the sentence emotionally positive? |
| 51. Does the sentence describe an activity related to daily life or routine? |
| 52. Does the text include a reference to a past era or time period? |
| 53. Does the input discuss a societal issue or social justice topic? |
| 54. Does the sentence convey a decision or choice made by the narrator? |
| 55. Does the sentence convey a sense of urgency or haste? |
| 56. Is the sentence providing an explanation or rationale? |
