# Supplementary material for: Interpretable embeddings of speech explain and enhance the brain encoding performance of audio models
Source: PLoS Comput Biol. 2026 Oct 30;22(10):e1014906. doi: 10.1371/journal.pcbi.1014906 (PMC13722164; doi:10.1371/journal.pcbi.1014906)
Supplement: Supplementary file 2 [file pcbi.1014906.s004.pdf]

S2 Table. Summary of the speech foundation models used in this study.

Model	# Layers	# Dimensions	# Parameters	Trainin
HuBERT X-large	48	1280	964M	Masked prediction of from iterative k-mean
WavLM Large	24	1024	317M	Same masked-unit obj + utterance-mixing an
Whisper Large	32	1280	638M	Supervised multilingu and speech-to-text tra
