# Supplementary material for: Interpretable embeddings of speech explain and enhance the brain encoding performance of audio models
Source: PLoS Comput Biol. 2026 Oct 30;22(10):e1014906. doi: 10.1371/journal.pcbi.1014906 (PMC13722164; doi:10.1371/journal.pcbi.1014906)
Supplement: Supplementary file 3 [file pcbi.1014906.s005.pdf]

S3 Table. Control for lexical predictability. Six word-level predictors of lexical predictability were added to the non-semantic control set: unigram log-frequency, bigram and trigram log-probability, skip-gram log-probability $\log P(w_t \mid w_{t-2})$, per-word GPT-2 surprisal, and the predictive entropy of the next-token distribution. The n-gram probabilities are from an interpolated trigram model counted over the Brown and Reuters corpora (2.5 M tokens); the GPT-2 values are the per-token probabilities released with the Podcast dataset. All six are aligned to the neural responses as the other word-level features are, by summing over words whose offset falls in $[t - 0.5, t]$ s. The table reports the proportion of the unique semantic variance in IFG pars triangularis accounted for by SFM representations (Fig 5e), computed with the baseline control set and with the lexical predictors added.

**Model**	**Layer**	**Baseline**	**+ Lexical**
Whisper Large	32	44.4%	39.9%
HuBERT X-large	32	36.7%	28.2%
WavLM Large	20	38.8%	31.5%
