# Supplementary material for: Interpretable embeddings of speech explain and enhance the brain encoding performance of audio models
Source: PLoS Comput Biol. 2026 Oct 30;22(10):e1014906. doi: 10.1371/journal.pcbi.1014906 (PMC13722164; doi:10.1371/journal.pcbi.1014906)
Supplement: Supplementary file 4 [file pcbi.1014906.s006.pdf]

S4 Table. Last-token versus within-step mean pooling of SFM representations. For each 250 ms step, the 12 SFM output frames (20 ms each) within the step were averaged instead of taking the final frame, with windows, context lengths, and regression settings otherwise identical. Values are shown at the layer used for each model in the main analyses: the ratio of shared to total SFM-explained neural variance (Fig 2b), and the test correlation averaged across electrodes for the SFM alone and for the joint model with interpretable features (Fig 3). Because both pooling methods were re-fitted with identical settings, the last-token values differ slightly from those in Fig 2b and Fig 3.

	**Shared ratio**		***r*, SFM alone**		***r*, joint model**	
**Model (layer)**	Last	Mean	Last	Mean	Last	Mean
Whisper Large (32)	0.787	0.725	0.0728	0.0760	0.0849	0.0878
HuBERT X-large (32)	0.850	0.800	0.0693	0.0721	0.0833	0.0856
WavLM Large (20)	0.809	0.755	0.0733	0.0776	0.0847	0.0869
