# Supplementary material for: Interpretable embeddings of speech explain and enhance the brain encoding performance of audio models
Source: PLoS Comput Biol. 2026 Oct 30;22(10):e1014906. doi: 10.1371/journal.pcbi.1014906 (PMC13722164; doi:10.1371/journal.pcbi.1014906)
Supplement: Supplementary file 5 [file pcbi.1014906.s014.pdf]

S1 Text. SFM feature extraction. Representations from speech foundation models (SFMs) were extracted using the code provided by Antonello et al. [1] (available at `https://github.com/HuthLab/encoding-model-scaling-laws`). The input audio file, totaling 1800 seconds, was divided into five contiguous 360-second segments before being fed into the SFMs. This segmentation was performed to prevent data leakage between training and test folds in the 5-fold cross-validation scheme.

References

1. Antonello RJ, Vaidya AR, Huth AG. Scaling laws for language encoding models in fMRI. In: Conference on Neural Information Processing Systems; 2023. .
