# Supplementary material for: Interpretable embeddings of speech explain and enhance the brain encoding performance of audio models
Source: PLoS Comput Biol. 2026 Oct 30;22(10):e1014906. doi: 10.1371/journal.pcbi.1014906 (PMC13722164; doi:10.1371/journal.pcbi.1014906)
Supplement: Supplementary file 6 [file pcbi.1014906.s015.pdf]

S2 Text. Effect of context length on brain prediction performance. Among the context sizes tested, the context size with the highest average correlation was 30 seconds for Whisper and HuBERT, and 5 seconds for WavLM (S3 Fig). This variability highlights the need for caution when interpreting the influence of context length on encoding performance. For instance, Anderson *et al.* found that EEG encoding performance with Whisper peaked at context lengths of 5–10 seconds and interpreted this as evidence that EEG preferentially reflects the encoding of speech within that temporal window. However, it is essential to distinguish whether such patterns reflect inherent properties of neural processing or are instead artifacts of model-specific biases.
