# Supplementary material for: Interpretable embeddings of speech explain and enhance the brain encoding performance of audio models
Source: PLoS Comput Biol. 2026 Oct 30;22(10):e1014906. doi: 10.1371/journal.pcbi.1014906 (PMC13722164; doi:10.1371/journal.pcbi.1014906)
Supplement: Supplementary file 7 [file pcbi.1014906.s016.pdf]

S3 Text. Variance partitioning with high-level features. In Fig 2c, the ratio for the phonetic, syntactic, and semantic features was computed using three-way variance partitioning among the SFM representations, speech probability, and the respective feature set (see S8 Fig), in order to control for speech presence information embedded within these higher-level features. For mel-spectrogram and Gabor filter bank features, no such control was applied, as they by definition represent the lowest-level confounds.
