# Supplementary material for: Interpretable embeddings of speech explain and enhance the brain encoding performance of audio models
Source: PLoS Comput Biol. 2026 Oct 30;22(10):e1014906. doi: 10.1371/journal.pcbi.1014906 (PMC13722164; doi:10.1371/journal.pcbi.1014906)
Supplement: Supplementary file 8 [file pcbi.1014906.s017.pdf]

We thank both reviewers for their careful and constructive reading. We are grateful to Reviewer 1 for the endorsement of the work, and to Reviewer 2 for pressing us on framing and controls. The comments prompted several new analyses that we believe materially strengthen the paper.

In brief, the revision adds a per-electrode map of encoding performance (S9 Fig); a control for lexical predictability, including surprisal, under which the semantic result is unchanged (S3 Table); a comparison of last-token and mean pooling; a replication of the main results at 8 and 16 Hz; a matched-context analysis addressing the temporal asymmetry between SFM and interpretable features; a full description of the semantic feature construction; an explicit statement of the correlational nature of the analyses in the Discussion; and public release of the code and semantic features.

Reviewer comments are reproduced in shaded blocks; our responses follow in plain text. Figure numbers refer to the rendered numbering of the revised manuscript.

Reviewer 1

We are grateful for the reviewer's positive assessment and for the thoughtout points they raised. We have treated each point as a request for clarification and have addressed all six.

Comment 1.1. How well is the encoding model working?

> Most of the plots do not show R or R2 but derived metrics like overlapping R2, unique R2 etc. And where they do show the empirical R/R2, it is averaged over all electrodes. Many of the prediction performance numbers are quite to very low. I understand this may be because of only looking at small partitions and because of averaging over many electrodes. But this is ECoG so surely there must be a few really high SNR channels where it works very well? It might be useful to show some visualisation with overall prediction performance (e.g. for different models) across electrodes to reassure a reader that the encoding model itself is working well? In particular because they use quite non-typical temporal alignment approach (no FIR, no epoching, but downsampling)?

Response.

The reviewer is right that the reported averages understate how well the encoding model performs on a subset of electrodes that are responsive to speech. The averages are taken over all 1,268 electrodes, which dilutes the mean substantially. The per-electrode distribution of the encoding correlation r is strongly right-skewed (Table R1).

Table R1: Distribution of the per-electrode encoding correlation r across all 1,268 electrodes, for each model at its best layer.

Model (best layer)	mean	median	p90	p99	max
Whisper-large, L32	0.073	0.024	0.249	0.592	0.726
HuBERT-xlarge, L32	0.069	0.025	0.228	0.564	0.697
WavLM-large, L20	0.067	0.022	0.225	0.553	0.693

To make this clear, we have added S9 Fig, which maps the per-electrode encoding correlation r onto a template brain for each model.

Comment 1.2. Negative partitions and the additivity assumption

> The authors write that negative R2s are "clipped". But they also report they use variance partitioning as in De Heer, Huth, et al. [...] that study also uses a "correction" method to deal with paradoxical results of combining cross-validation with variance partitioning [...] Do the authors also do this? If so, isn't this problematic given that many of their analyses hinge on overlapping partitions that are often very small on average? If not, and the authors simply clipped negative partitions, doesn't that equally create problems for the validity of the conclusions on many of the very small partitions? [...] More broadly, how do the authors think about the additivity assumption underlying their variance partitioning?

Response.

We thank the reviewer for this careful point, which led us to characterize our partitions far more thoroughly. We would like to answer this point in two steps.

(i) What we actually do.

Instead of clipping the shared or unique variance, we clip the *component* coefficients of determination (R^2_A, R^2_B, R^2_{AB}) at zero. We do so because a negative cross-validated R^2 is not a measurement of negative variance. It states that the fitted model, having absorbed noise in the training folds, predicts held-out data worse than the test-fold mean does ($SS_{res} > SS_{tot}$). Variance partitioning treats the components as shares of variance, and a negative share has no interpretation, so clipping maps "worse than nothing" onto "nothing".

Here, as a sanity check, we characterized which electrodes we are applying clipping to and examined whether the clipping zeroes electrodes that the encoding model does in fact predict well. Fig R1 plots each electrode's R^2 against that same model's encoding correlation r, for the interpretable features alone, the SFM embeddings alone, and the joint model. In all three the clipping is monotone in predictability: the clipped electrodes are confined to the low-correlation end of the distribution, and every electrode that the model predicts well is retained. The R^2 of the retained electrodes generally falls below r^2, which we would attribute to the ridge penalty biasing predictions toward zero.

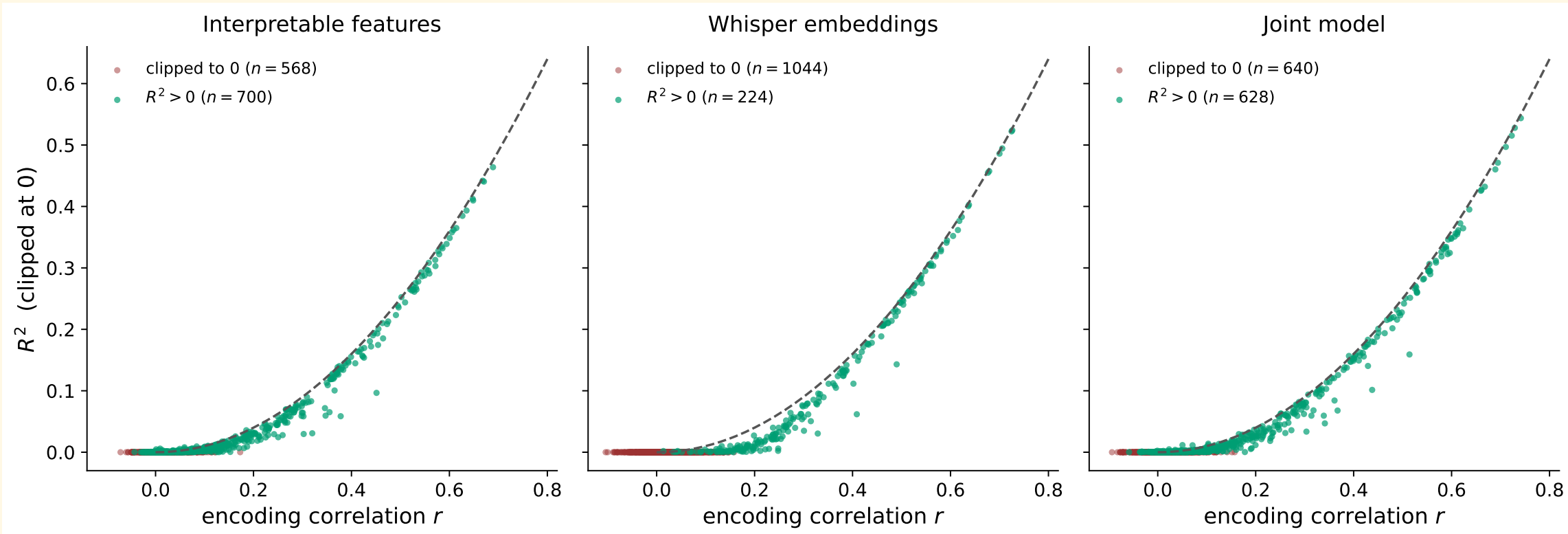

Figure R1: **Clipped electrodes are unpredictable ones.** Each point is one electrode, plotted as its R^2 against the encoding correlation r of the same model, at Whisper layer 32. Red points are electrodes clipped to zero, green points those with $R^2 > 0$. In every panel the clipped electrodes are confined to low correlations. The dashed curve is r^2.

The partitions are then derived from the clipped components:

$$\text{unique}_A = R^2_{AB} - R^2_B, \qquad \text{unique}_B = R^2_{AB} - R^2_A, \qquad \text{shared} = R^2_A + R^2_B - R^2_{AB}.$$

The derived partitions are *not* themselves clipped, and we do not apply the de Heer et al. additive bias correction. We note that retaining the negative values is the more conservative choice for our claims as clipping the shared partition to zero would inflate the ratio of shared variance.

(ii) Numerical quantification of errors by additivity violation.

Here, there are two types of additivity violation that should be treated differently.

A negative *shared* partition means $R^2_{AB} > R^2_A + R^2_B$, i.e. the joint model outperforms the sum of its parts (super-additivity). As the reviewer pointed out, the ratio $\text{shared}_i / R^2_{\text{SFM},i}$, where i denotes each electrode, would be unstable especially when the denominator is small. Fig R2 shows this instability. At Whisper layer 1, the per-electrode ratio is undefined at 1,025 of 1,268 electrodes, where R^2_{SFM} is exactly zero, and among the remaining 243 it ranges from -197.7 to $+11.6$. Notably, the median per-electrode ratio is 1.000 and the aggregate $\sum_i \text{shared}_i / \sum_i R^2_{\text{SFM},i}$ is 0.988 whereas the mean of per-electrode ratios is -0.053, a negative value for a quantity that should be bounded in $[0, 1]$. That is why in all relevant analyses of the ratio involving shared variance we calculate $\sum_i \text{shared}_i / \sum_i R^2_{\text{SFM},i}$, which weights each electrode by the variance it actually accounts for and is therefore far less prone to a small error in shared_i or $R^2_{\text{SFM},i}$.

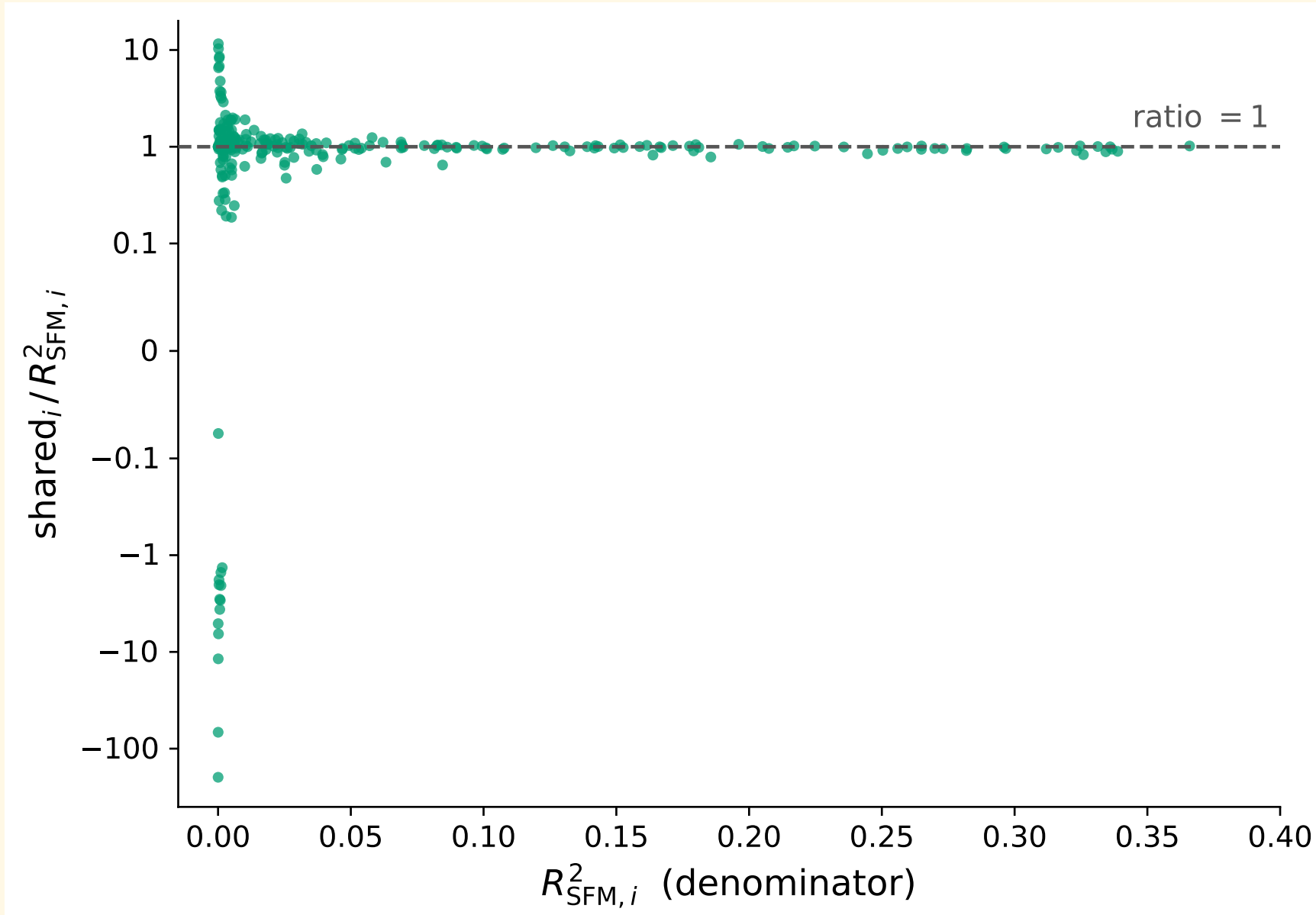

Figure R2: **The per-electrode shared-variance ratio is unstable where its denominator is small.** Each point is one electrode of Whisper layer 1 for which the ratio is defined ($n = 243$ of 1,268; R^2_{SFM} is exactly zero at the remaining 1,025). A well-behaved ratio would lie in $[0, 1]$; the dashed line marks the ceiling. Ratios scatter between -197.7 and $+11.6$ where the denominator is small, and collapse onto the ceiling once it exceeds roughly 0.05. The y axis is symmetric-log (linear within ± 0.1, logarithmic beyond).

An equivalent alternative would have been to keep the per-electrode ratio but introduce an exclusion criterion on the denominator. Table R2 shows that once electrodes with $R^2_{\mathrm{SFM}} < 0.05$ are removed, the two estimators agree to within 0.9% in all three models, whereas with no exclusion the mean of per-electrode ratios is displaced by more than 100% in two of them. Notably, the aggregate ratio itself barely changes under the cutoff by at most 0.4 percentage points. The discrepancy therefore lives entirely in the per-electrode estimates. We report the aggregate ratio because it requires no arbitrary threshold, but either choice supports the same conclusion.

Table R2: Two ways of forming the shared-variance ratio, at layer 1 of each model. "Mean" is the mean of per-electrode ratios $\frac{1}{n}\sum_i \mathrm{shared}_i / R^2_{\mathrm{SFM},i}$; "aggr." is the aggregate $\sum_i \mathrm{shared}_i / \sum_i R^2_{\mathrm{SFM},i}$ reported in Fig 2b; "diff." is their relative difference. Electrodes with $R^2_{\mathrm{SFM}} = 0$ are excluded throughout, as the per-electrode ratio is undefined there.

	no exclusion				$R^2_{\mathrm{SFM}} > 0.05$			
Model	n	mean	aggr.	diff.	n	mean	aggr.	diff.
Whisper	243	−0.053	0.972	105.5%	80	0.976	0.968	0.9%
HuBERT	248	0.918	0.938	2.1%	82	0.944	0.941	0.3%
WavLM	207	1.889	0.928	103.7%	81	0.929	0.932	0.3%

Separately, a negative *unique* partition means R^2_{AB} is below one of its own components, i.e.

the joint model is worse than a nested sub-model, a symptom of overfitting that motivates the de Heer correction. Fig R3 shows how large these violations are. No electrode falls short of its best component by more than 0.0094 in R^2, against gains reaching +0.047, and summed over all electrodes the shortfall comes to 0.98–1.34% of $\sum_i R^2_{AB,i}$ across the three models. As with the shared partition, we retain these negative values rather than clipping them, which is again the conservative choice for our claims.

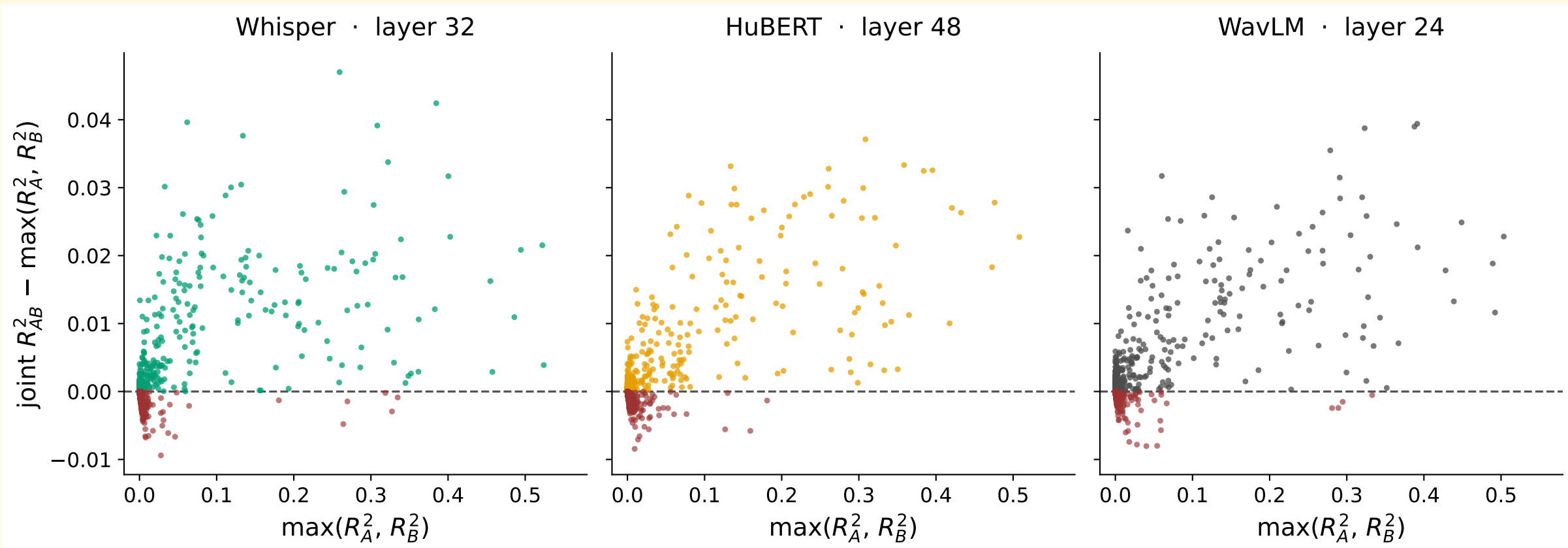

Figure R3: **The joint model dominates its components** For each electrode, the amount by which the joint model (interpretable features + SFM embeddings) exceeds the larger of its two component models, plotted against that component, at the final layer of each SFM. Points above the dashed line at zero are well behaved; points below it (red) are electrodes where the joint model is worse than a nested sub-model. Gains reach +0.047 in R^2 while no electrode falls short by more than 0.0094.

Comment 1.3. Surprisal and lexical predictability

> When isolating the "unique" neural variance of the GPT-4 semantic QA features, the baseline control spaces (syntax, phonetics, acoustics) entirely omit basic lexical properties like word frequency, n-gram probability, or standard LLM surprisal. Deep SFMs implicitly learn powerful language models, and the IFG strongly tracks lexical predictability. Without explicitly controlling for surprisal, isn't it likely that the shared variance attributed to these high-dimensional QA "semantics" is confounded by much simpler sequence predictability (and unigram, n-gram and skip-gram statistics)?

Response.

We agree this is an important control and decided to create additional features. Here, we built a set of six lexical features, covering each of the properties the reviewer names: unigram log-frequency, bigram and trigram log-probability, skip-gram log-probability ($\log P(w_t \mid w_{t-2})$), per-word GPT-2 surprisal, and the predictive entropy of the next-token distribution. The n-gram probabilities come from an interpolated trigram model counted over the Brown and Reuters corpora (2.5 M tokens). The GPT-2 values are the per-token probabilities released with the Podcast dataset itself, so the surprisal is that of the same model and tokenization used by Zada et al. All six are aligned to the brain responses exactly as the other word-level features are, by

summing over words whose offset falls in $[t - 0.5, t]$ s.

We then added these features to the non-semantic control set and repeated the same analysis used to produce Figure 5e in the manuscript (Table R3). Adding the lexical control reduces this "SFM Semantic Ratio" by between 4.5 and 8.5 percentage points depending on the model. A component of what the SFM representations account for is therefore attributable to lexical predictability, as the reviewer anticipates. Nevertheless, SFM representations continue to account for 28 to 40% of the unique semantic variance in the region, and the conclusion drawn in the manuscript, that SFMs encode brain-relevant semantic information but only partially, is unaffected.

Table R3: Proportion of the unique semantic variance in IFG pars triangularis accounted for by SFM representations (Fig 5e), computed with the baseline control set and with the lexical predictability control added. Each model is evaluated at the layer identified in Fig 2.

Model	layer	baseline	+ lexical
Whisper-large	32	44.4%	39.9%
HuBERT-xlarge	32	36.7%	28.2%
WavLM-large	20	38.8%	31.5%

These results, along with the definition and construction of the six lexical predictors, are now reported in S3 Table of the revised manuscript.

Comment 1.4. Why last-token rather than mean pooling?

> The authors use last-token pooling, citing earlier encoding model literature. However, the models they consider are not unidirectional autoregressive transformers that naturally pool information into the last token position. [...] in the speech model literature the standard and more appropriate choice is mean pooling, precisely because you otherwise discard information distributed across the sequence. I think it would be important to verify the main results with mean pooling; the current choice may systematically underestimate what these models encode.

Response.

We repeated the feature extraction with mean pooling over all valid output frames, at matched context, for the largest variant of each model, and re-ran the encoding analysis. Table R4 shows that mean pooling gives substantially worse encoding performance.

Table R4: Effect of mean pooling on the SFM representations. Encoding correlations r are averaged across all 1,268 electrodes, at matched context and at the layer identified for each model in Fig 2.

	Last token	Mean pooled
Adjacent-time-point cosine similarity	0.83–0.89	0.999
Whisper, r (SFM alone)	0.0728	0.0138
HuBERT, r (SFM alone)	0.0693	0.0196
WavLM, r (SFM alone)	0.0733	0.0137
Whisper, r (joint model)	0.0854	0.0788
HuBERT, r (joint model)	0.0832	0.0792
WavLM, r (joint model)	0.0850	0.0774

The reason is that mean pooling acts as a low-pass filter on the representation. Each sample is an average over all frames within the model's context window, and at a 250 ms hop consecutive windows share almost all of their frames, so the pooled vector changes very little from one sample to the next. The first row of Table R4 quantifies this: the cosine similarity between adjacent timepoints rises from 0.83–0.89 under last-token pooling to 0.999 under mean pooling. The representation is therefore close to constant in time, and a predictor that does not vary cannot track a time-varying neural response, which is why the encoding correlation of the SFM alone falls by 72 to 81%.

We would emphasise that this is not an argument that mean pooling discards information in general. It is the appropriate choice when a single representation is required for an utterance, as in most speech-model benchmarks, which is presumably why it is standard in that literature. The difficulty is specific to continuous encoding, where the predictor must vary on the timescale of the neural response. The same effect is evident in the joint model, whose correlation declines only slightly, from 0.0854 to 0.0788 for Whisper, because the interpretable features continue to supply the temporal structure that the pooled SFM representation has lost.

Comment 1.5. Why no time lags?

> In encoding modelling for continuous naturalistic data, people typically do either something like FIR (delays on the regressors) or epoching (many different predicted features). The authors use none of this and essentially deal with temporal alignment by using aggressive temporal smearing (concatenating features across 0.5 s windows at 4 Hz). I'm not suggesting they redo everything using a different linking function but I am curious what motivated the approach and if the authors aren't sacrificing a lot of the temporal granularity (and SNR) that ECoG stands out for?

Response.

The epoching approach the reviewer describes is standard in the literature mapping language models onto brain responses, where per-word features are regressed against neural activity time-locked to word onset with explicit lags. At the initial stage of designing our analyses, we concluded that this approach is not suitable for SFM embeddings and low-level speech features. Because SFM representations and acoustic features are continuous in time, generating per-word feature would require either averaging within each word or warping every word to a common length, which discard the temporal structure and have no physical basis. This is most obvious

for the mel-spectrogram, where "the spectrogram of a word" has no natural definition given that words in this stimulus span 0.02 to 1.26 s.

Our feature design is essentially an FIR basis, and we have modified the manuscript to make the equivalence clear. The mel-spectrogram predictor at time t comprises 32 mel bands at 21 consecutive frames spanning $[t - 0.5, t]$ s, so the design matrix contains one column for every band-by-delay pair. This is precisely an FIR design matrix with 21 delays at a 25 ms spacing, and the fitted weights constitute an impulse response relating the spectrogram to the neural response. The word-level predictors share the same 0.5 s temporal support but aggregate within it rather than resolving individual delays, since phonetic, syntactic and lexical events are sparse relative to the sampling grid. What distinguishes our implementation from a conventional FIR analysis is therefore the use of a single delay window common to all feature spaces, rather than a delay range selected separately for each predictor.

The 4 Hz sampling rate follows from the same reasoning. The word-level predictors cannot change faster than words do, and in this stimulus the median interval between word onsets is 220 ms, or 210 ms during connected speech, so a 250 ms sampling period is matched to the rate at which those predictors actually vary. To verify that our conclusions do not depend on the choice of the sampling rate, we repeated major analyses at 8 Hz and 16 Hz. Here, the results show that the conclusions still hold: the shared-variance ratio of Fig 2b changes only marginally (Table R5), a maximum deviation of 3.2% across a fourfold change in sampling rate.

Table R5: Shared-variance ratio of Fig 2b, $\sum_i \text{shared}_i / \sum_i R^2_{\text{SFM},i}$, at three neural sampling rates.

Model	layer	4 Hz	8 Hz	16 Hz
Whisper-large	32	0.790	0.778	0.769
HuBERT-xlarge	32	0.840	0.828	0.813
WavLM-large	20	0.812	0.795	0.786

The joint model of Fig 3 likewise outperforms the SFM alone for every model at every rate (Table R6). The absolute correlations decline as the sampling rate increases, for both the SFM alone and the joint model. This is expected each sample carries more noise at higher sampling rate.

Table R6: Encoding correlation r, averaged across all 1,268 electrodes, for the SFM representations alone and for the joint model with the interpretable features, at three neural sampling rates. Each model is evaluated at the layer identified in Fig 2.

		SFM alone			Joint model		
Model	layer	4 Hz	8 Hz	16 Hz	4 Hz	8 Hz	16 Hz
Whisper-large	32	0.0728	0.0648	0.0561	0.0854	0.0743	0.0617
HuBERT-xlarge	32	0.0693	0.0626	0.0543	0.0831	0.0723	0.0607
WavLM-large	20	0.0733	0.0656	0.0561	0.0850	0.0747	0.0616

Comment 1.6. Temporal asymmetry between SFM and interpretable features

> In principle, the transformers here can pool information across the entire context window. Doesn't this create a temporal asymmetry where the SFM models may explain unique variance because the feature is simply "slower" (uses more context) than the interpretable feature spaces, rather than actually capturing different information? Can the authors rule this out?

Response.

This is a fair concern and one we can address directly, because the variance partitioning was computed across seven SFM context lengths, corresponding to total input windows of 0.5, 1, 2.5, 5, 10, 20 and 30 s. The input window of 0.5 s matches the temporal window of the interpretable features (except for interpretable semantic features, which have longer windows), and thus provides the direct point of comparison. Table R7 shows that at matched context length, SFM embeddings still retain 42 to 55% of the unique variance it accounts for at its optimal context. Here, the effect remains significant in all three models (Wilcoxon signed-rank against zero, $p = 1.1 \times 10^{-9}$, 2.5×10^{-4} and 1.3×10^{-4} for Whisper, HuBERT and WavLM respectively).

Table R7: Unique neural variance accounted for by the SFM representations with respect to the combined interpretable feature set, averaged across all 1,268 electrodes, at the matched and at the optimal SFM context length. Each model is evaluated at the layer identified in Fig 2.

Model	layer	matched (0.5 s)	optimal	retained
Whisper-large	32	0.00223	0.00403 (30 s)	55%
HuBERT-xlarge	32	0.00105	0.00250 (30 s)	42%
WavLM-large	20	0.00181	0.00337 (5 s)	54%

We would not claim that longer temporal context is irrelevant. The unique variance does increase with context length, approximately doubling between the matched and the optimal setting, so a part of what the SFM contributes beyond the interpretable features is attributable to its longer integration window. We would emphasise, however, that integrating information over such windows is itself a capability of the SFM rather than a nuisance variable to be removed. The interpretable features cannot be extended to comparable extents in any practical way: preserving the resolution of the mel-spectrogram over a 30 s window would require $32 \times 1{,}201 \approx 38{,}000$ dimensions, well in excess of the 7,200 timepoints available. Compact long-range integration is precisely what the SFM supplies and the interpretable features do not, and we therefore regard the growth of unique variance with context as one of the substantive contributions of the SFM rather than as an artefact.

Reviewer 2

We thank the reviewer for a critical reading that has improved the framing of the paper considerably, particularly regarding our contribution relative to prior work and the interpretation of the variance partitioning.

Comment 2.1. Novelty relative to prior work

> The study's central claims—that SFM representations show layerwise progression from acoustic to higher-level linguistic information, that model–brain alignment improves with scale/context, and that interpretable features explain part of model performance—are closely related to existing findings in both brain-encoding and model-interpretability work (Antonello et al., 2023; Gao et al., 2025; Goldstein et al., 2025; Li et al., 2023). The authors should therefore clarify whether the present study offers a genuinely new conceptual account, or mainly repackages known layerwise representational trends using variance partitioning.

Response.

We have revised the Introduction and Discussion to state our contribution more precisely. The prior work the reviewer cites establishes that layerwise representational trends exist and that alignment scales with model size; our contribution is not to redescribe those trends but to *attribute* them. This distinction matters because the study was initially motivated by findings in the existing literature that seemed difficult to reconcile. Vaidya et al. (2022) report that low-level acoustic features explain no unique variance even when partitioned against the later layers of an SFM, while others disagree over whether SFMs carry brain-relevant semantic information at all (Anderson et al., 2024; Oota et al., 2024). By measuring SFM–brain alignment directly against an explicit basis of acoustic, phonetic, syntactic and semantic features, we identify what that alignment is attributable to rather than inferring it indirectly. Specifically: (i) we quantify how much of SFM–brain alignment is accounted for by known interpretable features, and find that it is the large majority, a question none of the cited studies address; (ii) we show that the layerwise progression is a *trade-off* rather than a monotonic accumulation, with brain-relevant low-level information actively lost as high-level information is gained; and (iii) we derive a practical consequence, namely that combining SFM embeddings with interpretable features yields a better and more interpretable encoding model than either alone. We hope that our analyses offer a common reference point against which claims about the representational basis of SFM–brain alignment can be evaluated.

Comment 2.2. Semantic feature construction

> The description of the semantic feature construction is not sufficiently clear. [. . .] The authors should describe this procedure in detail in the Methods section, including how the questions are presented to the model, how the answers are converted into feature vectors, and how these word-level features are aligned with the neural data. Rather than simply referring to a prior study, the manuscript should provide enough methodological detail for readers to understand and reproduce the semantic feature extraction procedure.

Response.

We thank the reviewer for raising this. The Methods now describe the construction of the semantic features in full, including the prompt presented to the model, the questions used, how the answers are converted into feature vectors at each context length, and how the resulting word-level vectors are aligned with the neural data.

Comment 2.3. Justification of 4 Hz and window lengths

> The neural data are downsampled to 4 Hz, and several features are constructed using windows such as [t - 0.5, t]. This choice may be reasonable, but the paper's claims about temporal integration windows and fine acoustic detail depend strongly on these preprocessing decisions. Could the authors justify the choice of 4 Hz downsampling and the specific temporal windows used for feature construction? It would also be helpful to report whether the main findings are robust to alternative downsampling rates, window lengths, or lag structures.

Response.

The 4 Hz rate follows from the rate at which the predictors themselves change. The word-level features cannot vary faster than words do, and in this stimulus the median interval between word onsets is 220 ms, or 210 ms during connected speech, so a 250 ms sampling period is matched to the rate at which those predictors actually vary.

On window lengths, the manuscript already contains the requested robustness analysis. The analysis behind Fig 4 computes shared variance against mel-spectrograms at seven window lengths spanning 0.025 to 0.5 s, four of which are shown in the figure, and the reported trade-off between temporal precision and window breadth is derived from exactly this sweep.

We have also verified that the findings do not depend on the sampling rate. We re-derived the high-gamma envelope and recomputed all feature representations at 8 Hz and at 16 Hz, and repeated both principal analyses. The shared-variance ratio of Fig 2b changes by at most 3.2% across this fourfold change in rate, and the joint model of Fig 3 continues to outperform the SFM alone for every model at every rate. Reviewer 1 raised the same question from the opposite direction, and the full comparison is reported in our response to comment 1.5 as Tables R5 and R6.

On lag structures, we set out in our response to comment 1.5 that the mel-spectrogram predictor is already an FIR design matrix with 21 delays spanning $[t - 0.5, t]$ s, and that the word-level predictors share the same temporal support.

Comment 2.4. Is the joint-model improvement trivial?

> The finding that adding hand-crafted acoustic, phonetic, syntactic, and semantic features improves encoding performance over a single SFM layer is not surprising, because the authors themselves argue that no single SFM layer preserves all relevant information. [...] The authors should show that the improvement is not merely due to adding more predictors or feature spaces, but reflects a principled and nontrivial gain.

Response.

Table 1 of the manuscript already reports the comparison the reviewer asks for, and we have made its logic explicit in the revised text. Both baselines there add a further feature space of 1,280 dimensions to the best SFM layer: Baseline 1 adds a second layer of the same model, and Baseline 2 adds the best layer of a different SFM. Our joint model adds the six interpretable feature spaces, 1,431 dimensions in total. The added capacity is therefore matched to within 12%, but the gains are not. Averaged across electrodes, Whisper improves from $r = 0.073$ to

0.075 under either baseline and to 0.085 with the interpretable features; HuBERT from 0.069 to 0.072 and 0.075 against 0.083; WavLM from 0.073 to 0.074 against 0.085. In every case the interpretable features produce a larger improvement than a second SFM layer or an entire second SFM, despite contributing a comparable number of dimensions.

Comment 2.5. Correlational interpretation

> The variance-partitioning framework is useful, but it remains correlational. Shared variance between SFM embeddings and interpretable features indicates overlap in predictive information, not that the SFM "uses" those features in a mechanistic sense or that the brain represents them in the same way. This is especially important because natural speech features are highly collinear.

Response.

We agree. Variance partitioning quantifies overlap in predictive information and warrants no stronger inference: shared variance between an SFM representation and an interpretable feature space does not establish that the model computes that feature, nor that the brain employs a corresponding representational scheme. We have added a paragraph to the Discussion making this explicit. It clarifies that we use *encode* throughout in the sense conventional to the encoding-model literature, namely that information about a property is linearly recoverable from a representation, rather than as an assertion about the computations that give rise to that representation or the use to which it is put. It further notes that adjudicating mechanistic claims would require interventional evidence, for instance ablating or steering model components and measuring the consequences for neural prediction.

Data and code availability

> *Reviewer 1:* They say we can access the code upon request. This is too bad and not in line with the policy.

Response.

We agree and apologise for the original statement. All code required to reproduce the analyses has been deposited in a public archive with the DOI 10.5281/zenodo.22296432, covering feature construction, the banded ridge encoding models, the variance partitioning and every figure in the manuscript. The underlying ECoG recordings and the stimulus are already publicly available as part of the Podcast ECoG Dataset (Zada et al., 2025), and the intermediate feature representations are regenerated from them by the deposited code. The one exception is the question-answering semantic features, which are produced through a commercial language model API and therefore cannot be reproduced exactly by re-running the pipeline; we have deposited those features directly, together with the per-word answers from which they are built. The Data Availability Statement has been updated accordingly.
