# Supplementary material for: Interpretable embeddings of speech explain and enhance the brain encoding performance of audio models
Source: PLoS Comput Biol. 2026 Oct 30;22(10):e1014906. doi: 10.1371/journal.pcbi.1014906 (PMC13722164; doi:10.1371/journal.pcbi.1014906)
Supplement: Supplementary file 9 [file pcbi.1014906.s018.pdf]

We thank the editor and both reviewers for their careful reading of the revised manuscript. In this revision, we have repeated the main analyses with the SFM frames averaged within each 250 ms step, as Reviewer 1 suggested, and report the results in a new supplementary table (S4 Table). We have also added a subsection to the Methods describing the temporal structure of the encoding model, and noted in the Discussion that other ways of pooling SFM representations can yield different results.

## Reviewer 1

---

> I'd like to thank the authors for their response which was mostly satisfactory, although a few more additions of e.g. figures to the paper of the type I'd asked for would have improved the study, I think. However, one point remain, in what I would consider a minor revision.

**Response.** We thank the reviewer for the careful and constructive review across both rounds. In this revision, the pooling analysis is included in the manuscript as S4 Table, alongside the per-electrode map of encoding performance (S9 Fig) and the control for lexical predictability (S3 Table) added in the previous round, and the temporal structure of the encoding model is now described in the Methods.

### Comment 1.1. Pooling within each 250 ms step

> Pooling. Perhaps my comment was not sufficiently specific. I did not, of course, intend "mean pooling" over the entire context window? I I meant averaging the frames within every 250 step, instead of taking the last embedding? I still think this is not the appropriate way to deal with these kinds of models for the reasons outlined in my prior review, and I don't think the authors analysis addresses it?

**Response.** We thank the reviewer for the clarification. We have now repeated the analysis with the pooling the reviewer describes: for each 250 ms step, we averaged the 12 SFM output frames (20 ms each) that fall within the step instead of taking the final frame, keeping the windows, context lengths and regression settings otherwise identical. Table R1 compares the two at the layer used for each model in the manuscript.

Table R1: Last-token versus within-step mean pooling of SFM representations, at the layer used for each model in the manuscript. "Shared ratio" is the ratio of shared to total SFM-explained neural variance (Fig 2b); $r$ is the test correlation averaged across electrodes for the SFM alone and for the joint model with interpretable features (Fig 3). Both pooling methods were re-fitted with identical windows and regression settings, so the last-token values differ slightly from those in Fig 2b and Fig 3.

| | | Shared ratio | | $r$, SFM alone | | $r$, joint model | |
|---|---|---|---|---|---|---|---|
| Model | layer | last | mean | last | mean | last | mean |
| Whisper-large | 32 | 0.787 | 0.725 | 0.0728 | 0.0760 | 0.0849 | 0.0878 |
| HuBERT-xlarge | 32 | 0.850 | 0.800 | 0.0693 | 0.0721 | 0.0833 | 0.0856 |
| WavLM-large | 20 | 0.809 | 0.755 | 0.0733 | 0.0776 | 0.0847 | 0.0869 |

Within-step averaging modestly improves SFM encoding performance, by 4 to 6%, so the reviewer is right that last-token pooling slightly underestimates what these models encode. The main results nevertheless hold. The large majority of the neural variance explained by the SFMs remains shared with the interpretable features (72 to 80%, compared with 79 to 85% under last-token pooling), and the joint model continues to outperform the SFM alone in all three models, by 12 to 19%. We now report these results in S4 Table, and we note in the Discussion that other ways of pooling SFM representations can yield different results.

### Comment 1.2. Regression model

> Regression model. For others to build on their analyses I think the regression model deserves more explanation. It differs from what others typically do – usually all regressors are entered with delays (e.g. refs 9 and 36), whereas here only the mel-spectrogram has delays and the neural data are downsampled to 4 Hz. Could the authors explain their choices a bit more and also include that in the paper?

**Response.** We agree, and we have added a paragraph describing the temporal structure of the encoding model to the Methods ("Temporal structure of the encoding model"). In our model, every feature at time $t$ summarizes the stimulus within the preceding 0.5 s, so the high gamma response at $t$ is related to stimulus information at lags between 0 and 0.5 s. The feature spaces differ in how this window is represented. The mel-spectrogram enters as its 21 frames within the window, which is equivalent to an FIR model with 21 delays at 25 ms spacing. The word-level features act as a single delay spanning 0 to 0.5 s, because words occur too sparsely (median interval between word onsets of 220 ms) for finer delays to be populated. The SFM representation at $t$ is computed from audio ending at $t$ and integrates the preceding context within the model.

We did not add explicit delays to every regressor, as in the studies the reviewer cites (Anderson et al., 2024; Li et al., 2023), for two reasons. First, adding $D$ delays multiplies the dimensionality of each feature space by $D$, which for SFM representations of 1,024 to 1,280 dimensions quickly becomes difficult to estimate from 7,200 time points. Second, the SFM representations already integrate past acoustic context, so delayed copies would largely duplicate information that the representation at $t$ already contains. The 4 Hz sampling rate matches the rate at which the word-level features change, and, as reported in our previous response, the main results are unchanged when the neural data and all features are recomputed at 8 and 16 Hz.

## Reviewer 2

> I thank the authors for their efforts in addressing my concerns. I am satisfied with the revisions and have no further concerns.

**Response.** We thank the reviewer for the careful review and constructive comments, which have substantially improved the manuscript.
