# Supplementary material for: Interpretable embeddings of speech explain and enhance the brain encoding performance of audio models
Source: PLoS Comput Biol. 2026 Oct 30;22(10):e1014906. doi: 10.1371/journal.pcbi.1014906 (PMC13722164; doi:10.1371/journal.pcbi.1014906)
Supplement: Peer review history

# Peer review: Decision Letter 0

**DOI:** 10.1371/journal.pcbi.1014906.r001

**Authors:** Daniel Bush, Yuanning Li

12 Aug 2026

Interpretable embeddings of speech explain and enhance the brain encoding performance of audio models

PLOS Computational Biology

Dear Dr. Shimizu,

Thank you for submitting your manuscript to PLOS Computational Biology. After careful consideration, we feel that it has merit but does not fully meet PLOS Computational Biology's publication criteria as it currently stands. Therefore, we invite you to submit a revised version of the manuscript that addresses the points raised during the review process.

Please submit your revised manuscript by Oct 11 2026 11:59PM. If you will need more time than this to complete your revisions, please reply to this message or contact the journal office at ploscompbiol@plos.org. When you're ready to submit your revision, log on to https://www.editorialmanager.com/pcompbiol/ and select the 'Submissions Needing Revision' folder to locate your manuscript file.

Please include the following items when submitting your revised manuscript:

* A letter that responds to each point raised by the editor and reviewer(s). You should upload this letter as a separate file labeled 'Response to Reviewers'. This file does not need to include responses to formatting updates and technical items listed in the 'Journal Requirements' section below.

* A marked-up copy of your manuscript that highlights changes made to the original version. You should upload this as a separate file labeled 'Revised Manuscript with Track Changes'.

* An unmarked version of your revised paper without tracked changes. You should upload this as a separate file labeled 'Manuscript'.

We look forward to receiving your revised manuscript.

Kind regards,

Yuanning Li

Academic Editor

PLOS Computational Biology

Daniel Bush

Section Editor

PLOS Computational Biology

**Journal Requirements:**

If the reviewer comments include a recommendation to cite specific previously published works, please review and evaluate these publications to determine whether they are relevant and should be cited. There is no requirement to cite these works unless the editor has indicated otherwise.

1) Please ensure that the CRediT author contributions listed for every co-author are completed accurately and in full.

At this stage, the following Authors/Authors require contributions: Riki Shimizu, Richard J Antonello, Chandan Singh, and Nima Mesgarani. Please ensure that the full contributions of each author are acknowledged in the "Add/Edit/Remove Authors" section of our submission form.

The list of CRediT author contributions may be found here: https://journals.plos.org/ploscompbiol/s/authorship#loc-author-contributions

2) We ask that a manuscript source file is provided at Revision. Please upload your manuscript file as a .doc, .docx, .rtf or .tex. If you are providing a .tex file, please upload it under the item type u2018LaTeX Source Fileu2019 and leave your .pdf version as the item type u2018Manuscriptu2019.

3) Please upload all main figures as separate Figure files in .tif or .eps format. For more information about how to convert and format your figure files please see our guidelines:

https://journals.plos.org/ploscompbiol/s/figures

4) Please upload a copy of Figure Fig 1-5 which you refer to in your text on pages 2, 6, 8, 10, and 11. Or, if the figure is no longer to be included as part of the submission please remove all reference to it within the text.

5) We notice that your supplementary Figures, and Tables are included in the manuscript file. Please remove them and upload them with the file type 'Supporting Information'. Please ensure that each Supporting Information file has a legend listed in the manuscript after the references list.

**Reviewers' comments:**

Reviewer's Responses to Questions

**Comments to the Authors:**

**Please note here if the review is uploaded as an attachment.**

Reviewer #1: Shimizu et al. present an encoding study, studying the ability of speech foundation models (SFMs) to predict brain responses in an open acess 30 min ECoG dataset. They sepcifically ask how much variance of the SFMs can be equally well accounted for by well characterised, interpretable features, and how this evolves over layers and across models. They find that the vast majority of SFM-brain alignment is driven by standard interpretable features. However, they also observe a layerwise representational trade-off, where deeper layers lose fine-grained acoustic precision but gain broader temporal integration and brain-relevant semantics, and that augmenting SFM embeddings with interpretable features yields the most accurate encoding models.

Overall, this is a great paper. They ask a timely question, use appropriate and state of the art methods to address it, and I'm happy that this type of NeuroAI work is sent to journal like PCB instead of a conference (which often publish slightly rushed papers). All my comments should be read as clarifications to improve the study, not to reject it -- I wholeheartedly endorse publication in the journal.

I just have a few questions that I think deserve some scrutiny and/or revision

[I chose "major revision" but only because the form forces me to make a choice; depending on the answers to the questions below, perhaps the revision is actually minor]

1. How well is the encoding model working?

Most of the plots do not show R or R2 but derived metrics like overlapping R2, unique R2 etc. And where they do show the empirical R/R2, it is averaged over all electrodes. Many of the prediction performance numbers are quite to very low. I understand this may be because of only looking at small partitions and because of averaging over many electrodes. But this is ECoG so surely there must be a few really high SNR channels where it works very well? It might be useful to show some visualisation with overall prediction performance (e.g. for different models) across electrodes to reassure a reader that the encoding model itself is working well? In particular because they use quite non-typical temporal alignment approach (no FIR, no epoching, but downsampling)?

2. What did the authors do with negative partitions? (Or: additivity of explained variance)

The authors write that negative R2s are "clipped". But they also report they use variance partitioning as in De Heer, Huth, et al, who pioneered this form of variance partitioning with regularised cross-validated encoding modelling. However, that study also uses a "correction" method to deal with paradoxical results of combining cross-validation with variance partitioning, where because of overfitting (or suppressor variables or any other effect where cross validated explained variance is non-additive), the combined model (A_and_B) can explain less variance than the sum of the separate models (A+B). This results in negative SHARED explained variance which is weird and uninterpretable.

De Heer, Huth, et al. "correction" can be regarded as a slightly hack fix where they learn a minimum "bias" term that they add to the empirical R2s to avoid no shared partition is negative. Do the authors also do this? If so, isn't this problematic given that many of their analyses hinge on overlapping partitions that are often very small on average? If not, and the authors simply clipped negative partitions, doesn't that equally create problems for the validity of the conclusions on many of the very small partitions? (And the ratios between them which will be even more sensitive to small distortions!)

More broadly, how do the authors think about the additivity assumption underlying their variance partitioning? Unique variance portions are uncontroversial, but several of the more bespoke overlapping partitions they analyse can behave quite differently than expected when strict additivity is violated (for critiques: see e.g. the blog by Diedrichsen: https://www.diedrichsenlab.org/BrainDataScience/variance_partitioning/index.htm)

To be clear, I don't think this is in any sense a reason for rejection. I think it is overall the right method for this type of question, but I want the authors to engage with these critiques because they are applying it a bit too loosely and uncritically at this point.

3. What about surprisal?

When isolating the "unique" neural variance of the GPT-4 semantic QA features, the baseline control spaces (syntax, phonetics, acoustics) entirely omit basic lexical properties like word frequency, n-gram probability, or standard LLM surprisal. Deep SFMs implicitly learn powerful language models, and the IFG strongly tracks lexical predictability. Without explicitly controlling for surprisal, isn't it likely that the shared variance attributed to these high-dimensional QA "semantics" is confounded by much simpler sequence predictability (and unigram, n-gram and skip-gram statistics)?

4. Why last token?

The authors use last-token pooling, citing earlier encoding model literature. However, the models they consider are not unidirectional autoregressive transformers that naturally pool information into the last token position. While a few neuroAI papers have adopted this convention, in the speech model literature the standard and more appropriate choice is mean pooling, precisely because you otherwise discard information distributed across the sequence. I think it would be important to verify the main results with mean pooling; the current choice may systematically underestimate what these models encode.

5. Why no time lags?

In encoding modelling for continuous naturalistic data, people typically do either something like FIR (delays on the regressors) or epoching (many different predicted features). The authors use none of this and essentially deal with temporal alignment by using aggressive temporal smearing (concatenating features across 0.5 s windows at 4 Hz). I'm not suggesting they redo everything using a different linking function but I am curious what motivated the approach and if the authors aren't sacrificing a lot of the temporal granularity (and SNR) that ECoG stands out for?

6. Isn't there a TEMPORAL asymmetry between SFM and interpretable features?

Despite my gripes about the last-token extraction operation and that it may be too temporally local, I also wonder how the authors think about the opposite point. In principle, the transformers here can pool information across the entire context window. Doesn't this create a temporal asymmetry where the SFM models may explain unique variance because the feature is simply "slower" (uses more context) than the interpretable feature spaces, rather than actually capturing different information? Can the authors rule this out? If not, how do they deal with this concern?

Reviewer #2: This study examines why speech foundation models (SFM) including Whisper, HuBERT, and WavLM align with brain responses during naturalistic speech comprehension. Using ECoG data from 9 patients listening to a 30-minute podcast, the authors show that much of the models’ brain-predictive power can be explained by interpretable acoustic, phonetic, syntactic, and semantic features. They further find a layerwise shift from low-level acoustic encoding in early layers to higher-level linguistic and semantic encoding in later layers, with later layers also capturing unique neural variance beyond interpretable features. Combining SFM embeddings with interpretable features improves encoding performance, suggesting a hybrid approach for building more accurate and interpretable brain encoding models.

Although the study addresses an interesting and timely question, I have several major concerns that, in my view, prevent publication in its current form.

1. The study’s central claims—that SFM representations show layerwise progression from acoustic to higher-level linguistic information, that model–brain alignment improves with scale/context, and that interpretable features explain part of model performance—are closely related to existing findings in both brain-encoding and model-interpretability work (Antonello et al., 2023; Gao et al., 2025; Goldstein et al., 2025; Li et al., 2023). The authors should therefore clarify whether the present study offers a genuinely new conceptual account, or mainly repackages known layerwise representational trends using variance partitioning.

Antonello, R. J., Vaidya, A. R., & Huth, A. G. (2023). Scaling laws for language encoding models in fMRI. Advances in Neural Information Processing Systems, 36, 21895–21907.

Gao, C., Ma, Z., Chen, J., Li, P., Huang, S., & Li, J. (2025). Increasing alignment of large language models with language processing in the human brain. Nature Computational Science, 5(11), 1080–1090.

Goldstein, A., Wang, H., Niekerken, L., Schain, M., Zada, Z., Aubrey, B., Sheffer, T., Nastase, S. A., Gazula, H., Singh, A., Rao, A., Choe, G., Kim, C., Doyle, W., Friedman, D., Devore, S., Dugan, P., Hassidim, A., Brenner, M., … Hasson, U. (2025). A unified acoustic-to-speech-to-language embedding space captures the neural basis of natural language processing in everyday conversations. Nature Human Behaviour, 9(5), 1041–1055.

Li, Y., Anumanchipalli, G. K., Mohamed, A., Chen, P., Carney, L. H., Lu, J., Wu, J., & Chang, E. F. (2023). Dissecting neural computations in the human auditory pathway using deep neural networks for speech. Nature Neuroscience, 26(12), 2213–2225.

2. The description of the semantic feature construction is not sufficiently clear. For example, are the semantic features for each word extracted by applying the 56 yes/no questions to that word, possibly with different contextual windows? The authors should describe this procedure in detail in the Methods section, including how the questions are presented to the model, how the answers are converted into feature vectors, and how these word-level features are aligned with the neural data. Rather than simply referring to a prior study, the manuscript should provide enough methodological detail for readers to understand and reproduce the semantic feature extraction procedure.

3. The neural data are downsampled to 4 Hz, and several features are constructed using windows such as [t − 0.5, t]. This choice may be reasonable, but the paper’s claims about temporal integration windows and fine acoustic detail depend strongly on these preprocessing decisions. Could the authors justify the choice of 4 Hz downsampling and the specific temporal windows used for feature construction? It would also be helpful to report whether the main findings are robust to alternative downsampling rates, window lengths, or lag structures.

4. The finding that adding hand-crafted acoustic, phonetic, syntactic, and semantic features improves encoding performance over a single SFM layer is not surprising, because the authors themselves argue that no single SFM layer preserves all relevant information. Since the interpretable features are explicitly designed to span multiple levels of speech representation, their combination with SFM embeddings may almost necessarily improve performance. The authors should show that the improvement is not merely due to adding more predictors or feature spaces, but reflects a principled and nontrivial gain.

5. The variance-partitioning framework is useful, but it remains correlational. Shared variance between SFM embeddings and interpretable features indicates overlap in predictive information, not that the SFM “uses” those features in a mechanistic sense or that the brain represents them in the same way. This is especially important because natural speech features are highly collinear. Acoustic, phonetic, lexical, syntactic, and semantic variables co-vary in continuous speech, making shared and unique variance difficult to interpret causally.

**********

**Have the authors made all data and (if applicable) computational code underlying the findings in their manuscript fully available?**

The PLOS Data policy requires authors to make all data and code underlying the findings described in their manuscript fully available without restriction, with rare exception (please refer to the Data Availability Statement in the manuscript PDF file). The data and code should be provided as part of the manuscript or its supporting information, or deposited to a public repository. For example, in addition to summary statistics, the data points behind means, medians and variance measures should be available. If there are restrictions on publicly sharing data or code —e.g. participant privacy or use of data from a third party—those must be specified.

Reviewer #1: **No:**  They say we can access the code upon request. This is too bad and not in line with the policy

Reviewer #2: Yes

**********

PLOS authors have the option to publish the peer review history of their article (what does this mean?). If published, this will include your full peer review and any attached files.

If you choose “no”, your identity will remain anonymous but your review may still be made public.

**Do you want your identity to be public for this peer review?**  For information about this choice, including consent withdrawal, please see our Privacy Policy.

Reviewer #1: No

Reviewer #2: No

[NOTE: If reviewer comments were submitted as an attachment file, they will be attached to this email and accessible via the submission site. Please log into your account, locate the manuscript record, and check for the action link "View Attachments". If this link does not appear, there are no attachment files.]

**Figure resubmission:**

**Reproducibility:**

?>

# Author comment: Author response to Decision Letter 1

**DOI:** 10.1371/journal.pcbi.1014906.r002

10 Sep 2026

Attachment Submitted filename: Response_to_Reviewers.pdf

# Peer review: Decision Letter 1

**DOI:** 10.1371/journal.pcbi.1014906.r003

**Authors:** Daniel Bush, Yuanning Li

1 Oct 2026

PCOMPBIOL-D-26-01024R1

Interpretable embeddings of speech explain and enhance the brain encoding performance of audio models

PLOS Computational Biology

Dear Dr. Shimizu,

Thank you for submitting your manuscript to PLOS Computational Biology. The reviewers are generally satisfied with the revisions, but some minor points remain to be addressed. We therefore invite the authors for a minor revision of the manuscript.

Please submit your revised manuscript by Oct 31 2026 11:59PM. If you will need more time than this to complete your revisions, please reply to this message or contact the journal office at ploscompbiol@plos.org. When you're ready to submit your revision, log on to https://www.editorialmanager.com/pcompbiol/ and select the 'Submissions Needing Revision' folder to locate your manuscript file.

Please include the following items when submitting your revised manuscript:

* A letter that responds to each point raised by the editor and reviewer(s). You should upload this letter as a separate file labeled 'Response to Reviewers'. This file does not need to include responses to formatting updates and technical items listed in the 'Journal Requirements' section below.

* A marked-up copy of your manuscript that highlights changes made to the original version. You should upload this as a separate file labeled 'Revised Manuscript with Track Changes'.

* An unmarked version of your revised paper without tracked changes. You should upload this as a separate file labeled 'Manuscript'.

If you would like to make changes to your financial disclosure, competing interests statement, or data availability statement, please make these updates within the submission form at the time of resubmission. Guidelines for resubmitting your figure files are available below the reviewer comments at the end of this letter.

As the corresponding author, your ORCID iD is verified in the submission system and will appear in the published article. PLOS supports the use of ORCID, and we encourage all coauthors to register for an ORCID iD and use it as well. Please encourage your coauthors to verify their ORCID iD within the submission system before final acceptance, as unverified ORCID iDs will not appear in the published article. Only the individual author can complete the verification step; PLOS staff cannot verify ORCID iDs on behalf of authors.

We look forward to receiving your revised manuscript.

Kind regards,

Yuanning Li

Academic Editor

PLOS Computational Biology

Daniel Bush

Section Editor

PLOS Computational Biology

**Journal Requirements:**

If the reviewer comments include a recommendation to cite specific previously published works, please review and evaluate these publications to determine whether they are relevant and should be cited. There is no requirement to cite these works unless the editor has indicated otherwise.

1) We notice that your supplementary Figures are included in the manuscript file. Please remove them and upload them with the file type 'Supporting Information'. Please ensure that each Supporting Information file has a legend listed in the manuscript after the references list.

**Reviewers' comments:**

Reviewer's Responses to Questions

**Comments to the Authors:**

**Please note here if the review is uploaded as an attachment.**

Reviewer #1: I'd like to thank the authors for their response which was mostly satisfactory, although a few more additions of e.g. figures to the paper of the type I'd asked for would have improved the study, I think.

However, one point remain, in what I would consider a minor revision.

1) Pooling. Perhaps my comment was not sufficiently specific. I did not, of course, intend "mean pooling" over the entire context window? I I meant averaging the frames within every 250 step, instead of taking the last embedding? I still think this is not the appropriate way to deal with these kinds of models for the reasons outlined in my prior review, and I don't think the authors analysis addresses it?

2) Regression model. For others to build on their analyses I think the regression model deserves more explanation. It differs from what others typically do -- usually all regressors are entered with delays (e.g. refs 9 and 36), whereas here only the mel-spectrogram has delays and the neural data are downsampled to 4 Hz. Could the authors explain their choices a bit more and also include that in the paper?

Reviewer #2: I thank the authors for their efforts in addressing my concerns. I am satisfied with the revisions and have no further concerns.

**********

**Have the authors made all data and (if applicable) computational code underlying the findings in their manuscript fully available?**

The PLOS Data policy requires authors to make all data and code underlying the findings described in their manuscript fully available without restriction, with rare exception (please refer to the Data Availability Statement in the manuscript PDF file). The data and code should be provided as part of the manuscript or its supporting information, or deposited to a public repository. For example, in addition to summary statistics, the data points behind means, medians and variance measures should be available. If there are restrictions on publicly sharing data or code —e.g. participant privacy or use of data from a third party—those must be specified.

Reviewer #1: Yes

Reviewer #2: Yes

**********

PLOS authors have the option to publish the peer review history of their article (what does this mean?). If published, this will include your full peer review and any attached files.

If you choose “no”, your identity will remain anonymous but your review may still be made public.

**Do you want your identity to be public for this peer review?**  For information about this choice, including consent withdrawal, please see our Privacy Policy.

Reviewer #1: No

Reviewer #2: **Yes:**  Jixing Li

[NOTE: If reviewer comments were submitted as an attachment file, they will be attached to this email and accessible via the submission site. Please log into your account, locate the manuscript record, and check for the action link "View Attachments". If this link does not appear, there are no attachment files.]

**Figure resubmission:**

**Reproducibility:**

To enhance the reproducibility of your results, we recommend that authors of applicable studies deposit laboratory protocols in protocols.io, where a protocol can be assigned its own identifier (DOI) such that it can be cited independently in the future. Additionally, PLOS ONE offers an option to publish peer-reviewed clinical study protocols. Read more information on sharing protocols at https://plos.org/protocols?utm_medium=editorial-email&utm_source=authorletters&utm_campaign=protocols

# Author comment: Author response to Decision Letter 2

**DOI:** 10.1371/journal.pcbi.1014906.r004

7 Oct 2026

Attachment Submitted filename: Response_to_Reviewers_auresp_2.pdf

# Editor report: Decision Letter 2

**DOI:** 10.1371/journal.pcbi.1014906.r005

**Authors:** Daniel Bush, Yuanning Li

15 Oct 2026

Dear Mr. Shimizu,

We are pleased to inform you that your manuscript 'Interpretable embeddings of speech explain and enhance the brain encoding performance of audio models' has been provisionally accepted for publication in PLOS Computational Biology.

Before your manuscript can be formally accepted you will need to complete some formatting changes, which you will receive in a follow up email. A member of our team will be in touch with a set of requests.

Please note that your manuscript will not be scheduled for publication until you have made the required changes, so a swift response is appreciated.

IMPORTANT: The editorial review process is now complete. PLOS will only permit corrections to spelling, formatting or significant scientific errors from this point onwards. Requests for major changes, or any which affect the scientific understanding of your work, will cause delays to the publication date of your manuscript.

Should you, your institution's press office or the journal office choose to press release your paper, you will automatically be opted out of early publication. We ask that you notify us now if you or your institution is planning to press release the article. All press must be co-ordinated with PLOS.

Thank you again for supporting Open Access publishing; we are looking forward to publishing your work in PLOS Computational Biology.

Best regards,

Yuanning Li

Academic Editor

PLOS Computational Biology

Daniel Bush

Section Editor

PLOS Computational Biology

***********************************************************

# Editor report: Acceptance letter

**DOI:** 10.1371/journal.pcbi.1014906.r006

**Authors:** Daniel Bush, Yuanning Li

PCOMPBIOL-D-26-01024R2

Interpretable embeddings of speech explain and enhance the brain encoding performance of audio models

Dear Dr Shimizu,

I am pleased to inform you that your manuscript has been formally accepted for publication in PLOS Computational Biology. Your manuscript is now with our production department and you will be notified of the publication date in due course.

The corresponding author will soon be receiving a typeset proof for review, to ensure errors have not been introduced during production. Please review the PDF proof of your manuscript carefully, as this is the last chance to correct any errors. Please note that major changes, or those which affect the scientific understanding of the work, will likely cause delays to the publication date of your manuscript.

Soon after your final files are uploaded, unless you have opted out, the early version of your manuscript will be published online. The date of the early version will be your article's publication date. The final article will be published to the same URL, and all versions of the paper will be accessible to readers.

For Research, Software, and Methods articles, you will receive an invoice from PLOS for your publication fee after your manuscript has reached the completed accept phase. If you receive an email requesting payment before acceptance or for any other service, this may be a phishing scheme. Learn how to identify phishing emails and protect your accounts at https://explore.plos.org/phishing.

Thank you again for supporting PLOS Computational Biology and open-access publishing. We are looking forward to publishing your work!

With kind regards,

Anitha Samidurai

PLOS Computational Biology | Carlyle House, Carlyle Road, Cambridge CB4 3DN | United Kingdom ploscompbiol@plos.org | Phone +44 (0) 1223-442824 | ploscompbiol.org | @PLOSCompBiol
